# Catastrophic health expenditure and its determinants in Kenya slum communities

**DOI:** 10.1186/s12939-015-0168-9

**Published:** 2015-05-14

**Authors:** Steven Buigut, Remare Ettarh, Djesika D Amendah

**Affiliations:** American University in Dubai, School of Business, P.O. Box 28282, Dubai, UAE; African Population Health Research Center, APHRC Campus, Kirawa Road, P.O. Box 10787–00100, Nairobi, Kenya

## Abstract

**Background:**

In Kenya, where 60 to 80% of the urban residents live in informal settlements (frequently referred to as slums), out-of-pocket (OOP) payments account for more than a third of national health expenditures. However, little is known on the extent to which these OOP payments are associated with personal or household financial catastrophe in the slums. This paper seeks to examine the incidence and determinants of catastrophic health expenditure among urban slum communities in Kenya.

**Methods:**

We use a unique dataset on informal settlement residents in Kenya and various approaches that relate households OOP payments for healthcare to total expenditures adjusted for subsistence, or income. We classified households whose OOP was in excess of a predefined threshold as facing catastrophic health expenditures (CHE), and identified the determinants of CHE using multivariate logistic regression analysis.

**Results:**

The results indicate that the proportion of households facing CHE varies widely between 1.52% and 28.38% depending on the method and the threshold used. A core set of variables were found to be key determinants of CHE. The number of working adults in a household and membership in a social safety net appear to reduce the risk of catastrophic expenditure. Conversely, seeking care in a public or private hospital increases the risk of CHE.

**Conclusion:**

This study suggests that a substantial proportion of residents of informal settlements in Kenya face CHE and would likely forgo health care they need but cannot afford. Mechanisms that pool risk and cost (insurance) are needed to protect slum residents from CHE and improve equity in health care access and payment.

## Introduction

Catastrophic health expenditure (CHE) occurs when out-of-pocket (OOP) payments for health services consume such a large portion of a household’s available income and the household may be pushed into poverty as a result [1]. In situations where health financing mechanisms that protect households against the financial risks associated with ill health are unavailable or deficient, even modest healthcare bills could lead to CHE. In addition to financial shock from medical expenses for treatment, households are often faced with income loss if affected members are working adults. The possibility that CHE leads households into impoverishment thus raises equity concerns [1-3].

In Kenya, national level data indicate that household OOP payments accounted for 54% and 37% of the total health expenditure in 2002 and 2010–11, respectively [4]. While the more recent level is much lower than about a decade earlier, it still represents a high proportion of total health expenditure and suggests that some households may be paying substantial amounts for health care. This is particularly important as national insurance coverage in Kenya is low: only 7% of women aged 15–49 years had health insurance, which was mainly provided by employers [5]. Insurance coverage among men is unlikely to be significantly different from that of women as most insurance schemes tend to cover families including spouses and children. Among women age 15–49 in Kenya, only 31% have never been married [6].

The above proportions of OOP in the total health expenditures in the country and low rates of insurance are likely to conceal disparities across rural and urban areas, and even within urban areas. Disparities and inequity in access and utilization of health care as well as health outcomes have been documented between rural and urban areas [6] and within urban areas [7] in Kenya and elsewhere in sub-Saharan Africa. Such differences are likely to translate into differences in OOP.

In Kenya, about a quarter of the population lives in urban areas [6]. Among them, 60% to 80% of urban residents live in slum or slum like conditions [8] characterized by a higher level of unemployment, general deprivation including monetary poverty, and poorer health indicators than the city average [7]. Such figures are alarming as 55% of the population of sub-Saharan Africa is projected to be living in urban areas by 2050 [9]. However, there is a dearth of knowledge on slum dwellers as they are often excluded from national surveys. This is because their dwelling areas are often considered illegal and do not appear on official enumerations areas used to draw samples, or the data for slums are aggregated with that of other non-slum areas of cities [10]. Information specific to slum areas might be useful for addressing residents’ health-related issues as those populations should not be ignored in policy making.

This study investigates the incidence and determinants of CHE among slum communities in Kenya between May 2012 and April 2013. It applies several approaches in an attempt to tease out a consistent set of household characteristics related to catastrophic health expenditure.

### Approaches to catastrophic health expenditures

Catastrophic health expenditure is commonly described as a health care budget share that exceeds a pre-defined threshold [11]. Any such cut-off is necessarily fraught with problems and no firm consensus exists on the thresholds in literature [12].

Two approaches are frequently applied in the literature [2]. The first approach sets the threshold in terms of proportionality of income. This approach considers the OOP payments as a proportion of income (X). That is (OOP/X). Thresholds used varied from 2.5% to 15%. However, using the same threshold for both the poor and rich households is problematic for equity reasons as richer households are more likely to exceed the threshold level with less adverse effect than the poor ones especially at higher thresholds levels [2].

The second approach is based on ability-to-pay. This approach considers OOP payments in terms of a measure of ability to pay (y), such that (OOP/y) where y = X-S_exp_. The S_exp_ is subsistence deductions, while X is income as indicated in the first approach above (or consumption expenditure). Expenses allowed in S_exp_ to compute the ability to pay has been a subject of debate in the literature. For example, some studies compute ability to pay as income less actual food spending [2]. However household food expenditure may not capture actual subsistence expenditure as food spending by higher income households may include non-essential food. To overcome this limitation, a method proposed by WHO [12] expresses capacity to pay as effective income remaining after basic subsistence. Subsistence expenditure (S_exp_) is defined as the average food expenditure of households whose food expenditure share is in the 45th to 55th range. Hence y = X-S_exp, 45th/55th_, with X as consumption expenditure. This methodology has been slightly modified by considering all necessities rather than food consumption only [13].

To allow for international comparability, while excluding non-essential spending, the subsistence level could be based on some internationally recognised cut-off such as the dollar-a-day poverty line used by the World Bank [14,15]. Note that there is a push for the revision of this poverty line to USD 1.25 dollars a day [16]. Like other measures, the use of a poverty line value such as the dollar a day cut-off, also has limitations. For example it introduces uncertainty arising from the construction of food purchasing power parity (PPP) conversion factors [14]. Using a flat rate deduction poses the additional problem that capacity to pay (y) could become zero or negative, leading to an undefined or negative ratio.

More recently a number of researchers have used a methodology proposed by the World Health Organization (WHO) [1] to compute the subsistence expenditure and the catastrophic health spending and impoverishment. This methodology incorporates an approach that circumvents the weakness related to estimation of PPP inherent to the use of an international poverty line and also avoids the problem of negative capacity to pay. This WHO methodology uses a food share-based poverty line for estimating subsistence. In this approach the poverty line is defined as the food expenditure of the household whose food expenditure share of total household expenditure is at the 50th percentile. Steps used to identify catastrophic health spending have been detailed elsewhere [1,17].

### Determinants of catastrophic health expenditures

The literature suggests that a wide range of household characteristics affect the probability of incurring catastrophic health expenditure. For example, availability of health insurance reduces the likelihood of occurrence of CHE [12,15,17]. On the contrary, households with hospitalised members, with elderly, or chronically ill members [18], and those who use in-patient service especially private hospitals [3] are more likely to face CHE. Other factors that increase the likelihood of CHE are: age of head of household, children in the household, gender of the household head, and level of education. Moreover, results can be sensitive to methodology and definitions of key indicators such as the OOP expenditure [13]. A study on how households cope with OOP health expenditures in 15 African countries found that in most of these countries, health financing is too weak to provide protection for households from health shocks [19]. Thus, borrowing and depletion of assets to finance health care was prevalent among the lower income quintiles. In the only study to date using Kenya data, incidence of CHE varied with the methods and the threshold used [20]. For example, about 10% of households incurred a catastrophic expenditure with the threshold of 25% when computation is based on the total expenditures. That proportion increased to 16% when the authors used only the non-food expenditure. The study also indicated that for any given method or threshold used, the proportion of household facing catastrophic health expenditures decreased with the quintile of wealth, raising equity questions.

## Methods

### Study setting

This paper uses unique data from the Indicator Development for Surveillance of Urban Emergencies (IDSUE) project implemented by the African Population and Health Research Center (APHRC) in partnership with Concern Worldwide (Kenya). The objective of the project was to develop early warning indicators to identify slow-onset humanitarian emergencies in urban slums. The study was conducted in Nairobi (four slums: Viwandani, Korogocho, Dandora, and Mukuru) and Kisumu, a city 265 km west of Nairobi (two slums: Obunga and Nyalenda).

Although the individual slum communities were unique in some respects, the common characteristics included poor and unsafe dwelling structures, lack of access to piped water, poor environmental sanitation, high unemployment and low incomes, low education levels, and high disease prevalence. Viwandani slum is located very close to the Nairobi’s industrial area and is populated predominantly by young male adults working in the nearby factories and who have migrated from rural areas without their spouses. Korogocho is a larger and older slum settlement with a higher proportion of the elderly and families that have resided there for decades [21]. Mukuru slum is located near Nairobi's industrial area and is comprised of residents who work as casual labourers or as petty traders hawking various items. Malaria, HIV/AIDS, typhoid and dysentery are prevalent in Mukuru, and are likely linked to the poor sanitation in the slum [22]. Dandora, is a high-density slum located near Korogocho, and is home to the largest dumpsite in Nairobi. The dumpsite is a major source of pollutants and toxic chemicals through the air and ground water causing respiratory, gastrointestinal and dermatological illnesses among residents [23].

The Nyalenda and Obunga slums are the largest in Kisumu with population densities of between 6,000–8,000 people per square kilometer. Many of the residents in both slums do not have access to piped water, electricity or adequate toilet facilities. Poverty levels are high with 65%–78% of households in Nyalenda, and 55% of households in Obunga, classified as below the urban poverty line [24].

### Ethical consideration

Ethical approval for the IDSUE study was obtained from the Kenya Medical Research Institute (KEMRI) Ethical Review Committee. In all households included in the study, the head of the household (or his/her representative) was first approached to obtain consent to the participating in the interview. All the participants who consented to participate confirmed this by signing a written consent form. A resident respondent knowledgeable about the household finances and other affairs was then interviewed.

### Sampling methods

The household survey for the IDSUE project was first conducted in March 2011 and included seven rounds of data collection as of April 2013. The study collected data on residents (resident defined as a member of a household with minimum continuous stay of three months in the slum) using a household level survey conducted through an interviewer-administered questionnaire. For the present study, we used data from survey Rounds 4 to 7 which collected detailed health expenditures, using the same sampling methods and questionnaires. Earlier survey rounds did not include questions on health expenditure.

In the absence of population enumeration listings, households included in this study were randomly selected using a modified cluster sampling based on segmentation of villages. Each village was further broken down into segments of approximately equal size and the segments were all numbered. A random sample of the segments was taken and from each of the selected segments, all the households were listed and a random sample of the households to interview was taken from each selected segment. Other details on the sampling methods and the Nairobi Urban Health and Demographic Surveillance Systems can be found elsewhere [25] [26].

### Data collection and construction of main variables

#### Data collection

Questionnaires for Rounds 4 to 7 include detailed expenditure and income questions for all household members. In addition, the questionnaire sought to capture information related to food security, water and sanitation, household livelihoods, coping strategies, personal and property security, and food and non-food consumption. Expenditure and income data were collected in Kenya Shillings (KES). The average exchange rate during the survey period, May 2012 and April 2013, was 85.5KES per US dollar.

#### Main variables construction

The questionnaire collected the income of the household members with earnings in the preceding four weeks. For earners who get daily or weekly income, we adjusted to a monthly value with the appropriate multiplier (26 or 4 respectively). We aggregated the earnings of all workers to obtain the household income. This household income was divided into tertiles. The expenditure data collection was item specific with appropriate reference periods. When reference periods were different, expenditures were all consolidated to cover the same unit of four weeks. For instance, utilities expenditures reference period was the last month before the interview while that of other clothing items were the three months before the interview to account for the fact that utility bills are monthly and clothing pieces are bought on a less regular basis. Thus, we divided the clothing expenditure by 3. Special care was taken to collect accurate information on food. First, details of food consumption for the previous day were collected, and then the total food expenditure information was collected for the seven days prior to the interview. For this article, the weekly expenditure on food was extrapolated to four weeks. Health expenditure collected includes spending on medicines, transport to and from health facilities, consultation and treatment costs, laboratory test and diagnostic fees, hospitalization fees, cost of visits to traditional healers, and other health related expenditures during the last three months converted to four weeks.

### Empirical methodology

We use two approaches to define CHE. First, we use the ability to pay approach to determine CHE following the WHO method [1]. The steps in this approach to identify catastrophic health spending have been detailed elsewhere [1,17] and are summarized below. The results based on this approach are presented as Model 1A and 1B in Tables 1 and Model 1A in Table 2.

**Table 1:** **Proportion of households experiencing catastrophic health expenditure**

**Indicators**	*Model 1A: WHO capacity to pay approach	**Model 1B: WHO capacity to pay approach**	**Model 2: OOP as percent of household income**
Catastrophic 30	144^1^ [1.52%]^2^ (0.123)^3^	146 [1.55%] (0.123)	1745 [18.46%] (0.388)
Catastrophic 20	254 [2.69%] (0.162)	255 [2.70%] (0.162)	1860 [19.67%] (0.398)
Catastrophic 15	383 [4.05%] (0.197)	388 [4.11%] (0.198)	1969 [20.83%] (0.406)
Catastrophic 10	571 [6.04%] (0.238)	575 [6.09%] (0.239)	2155 [22.80%] (0.420)
Catastrophic 05	-	-	2683 [28.38%] (0.451)
Subsistence expenditure	{6439.4}^4^ (1400.4)	{6591.9} (1943.9)	-
Mean capacity to pay	{11549.5} (12126.9)	{11513.6} (120049.2)	-
Poor household	644 [6.8%] (0.252)	697 [7.37] (0.261)	-

Notes: ^1^Indicates the number of households reporting the event. ^2^Placed in [..] is the percentage of households reporting CHE. ^3^Placed in (..) is the standard deviation. ^4^Placed in {..} is the mean of the stated row variable.

Models 1 is based on the Xu [1] procedure described in the text with varying level of household scale multiplier. *Model 1A uses household scale multiplier β = 0.41, and 45^th^ – 55^th^ percentile to compute subsistence expenditure. Model 1B household scale multiplier β = 0.56, and 45^th^ – 55^th^ percentile to compute subsistence expenditure.

**Table 2:** **Determinants of CHE based on the WHO’s Xu** [1] **approach**

	**Model 1A**		
**Variable (N = 8171)**	**10%**	**20%**	**30%**
**Round:**			
Round 4 (Ref)			
Round 5	0.89^a^ (0.19)^b^	0.88 (0.28)	0.96 (0.44)
Round 6	1.25 (0.28)	1.16 (0.38)	1.31 (0.63)
Round 7	0.88 (0.20)	0.81 (0.27)	1.02 (0.49)
**Slum:**			
Viwandani (Ref)			
Korogocho	1.01 (0.16)	1.09(0.24)	1.64 (0.52)
Dandora	0.99 (0.23)	0.64(0.23)	0.95 (0.45)
Mukuru	1.09 (0.17)	0.71(0.18)	0.71 (0.26)
Obunga	0.57 (0.11)***	0.61 (0.17)*	0.68 (0.26)
Nyalenda	0.68 (0.12)**	0.43 (0.12)***	0.36 (0.15)**
**Income** ^**1**^ **:**			
Lower tertile (Ref)			
Middle tertile	0.91 (0.12)	0.72 (0.14)*	0.87 (0.24)
Top tertile	0.84(0.11)	0.85 (0.16)	1.25 (0.33)
**Expenditure:**			
Lower tertile (Ref)			
Middle tertile	0.87 (0.11)	0.86 (0.18)	0.88 (0.26)
Top tertile	1.32 (0.18)**	1.85 (0.36)***	2.66 (0.70)***
**Livelihood:**			
Petty trade, casual (Ref)			
Formal labor	0.70 (0.10)***	0.70 (0.14)*	0.44 (0.14)***
Own business	0.73 (0.11)**	0.65 (0.15)*	0.39 (0.13)***
**Main bread winner is male**	1.11 (0.12)	1.05 (0.17)	1.24 (0.28)
**Working adult in household**	0.82 (0.10)	0.54 (0.11)***	0.51 (0.14)**
**Child under 5 years**	1.07 (0.12)	0.89 (0.15)	0.71 (0.16)
**Age of main income earner:**			
35 < =Age (Ref)			
35 < Age < =55	1.07 (0.13)	1.08 (0.19)	1.17 (0.28)
Age > 55	1.56 (0.32)**	2.11 (0.56)***	1.87 (0.68)*
**Duration of stay in slum**	1.02 (0.01)***	1.02 (0.01)**	1.02 (0.01)*
**Safety net**	0.63 (0.08)***	0.60 (0.11)***	0.50 (0.13)***
**Shocks**	1.08 (0.14)	1.17 (0.23)	0.97 (0.27)
**Type of illness**			
Other (Ref)			
Diarrhea	0.93 (0.19)	0.76 (0.25)	0.69 (0.35)
Fever	1.05 (0.16)	1.15 (0.26)	0.60 (0.20)
Cough	0.73 (0.11)**	0.45 (0.12)***	0.67 (0.23)
Headache	0.82 (0.12)	0.90 (0.20)	1.15 (0.36)
Vomiting	1.27 (0.26)	1.03 (0.34)	0.78 (0.41)
Seizure	1.80 (0.77)	1.71 (0.99)	2.23(1.52)
Difficult breathing	1.31 (0.29)	1.45 (0.46)	1.00 (0.44)
Measles	1.27 (0.55)	2.42 (1.23)*	1.88 (1.44)
Injury	2.15 (0.57)***	2.68 (0.91)***	2.05 (0.95)
**Care sought for illness**			
No ill person/no care sought (Ref)			
Ill/no care sought	1.99 (0.47)***	2.96 (0.93)***	1.24 (0.78)
Ill/care sought	1.82 (0.27)***	1.70 (0.37)**	1.96 (0.59)**
**Place care was sought:**			
Other (Ref)			
Public hospital	3.96 (0.71)***	4.24 (1.07)***	6.61 (2.14)***
Public clinic	0.66 (0.16)*	0.72 (0.27)	0.65 (0.36)
Private hospital	4.07 (0.99)***	2.87 (1.06)***	3.41 (1.61)***
Private clinic	1.73 (0.40)**	1.78 (0.61)*	1.72 (0.78)
Mission hospital	2.82 (1.33)**	3.35 (2.01)**	1.23 (1.32)
Mission clinic	0.95 (0.37)	0.76 (0.48)	1.01 (0.78)

Notes: (^a^) Reports the odds ratio. (^b^) Reports the standard error. *, **, *** indicate significance at 10, 5, and 1% level respectively.

In this Model 1A we use a household multiplier β = 0.41, and the 45^th^ – 55^th^ percentile to compute the subsistence expenditure. (^2^) The correlation between income group and expenditure group variables is 0.24.

#### WHO capacity to pay approach

The variables (listed below) and computational steps are to generate them are summarised here:

#### Variables

- FES_h_ = Food expenditure share for household
- FE_h_ = Food expenditure of household
- TE_h_ = Total expenditure of household
- HES = Household equivalent size
- HS = Household size
- β = This is the household scale multiplier. Two values are used (0.41 and 0.56).
- EFE_h_ = Equivalent food expenditure of household.
- PL = poverty line
- SE_h_ = Subsistence expenditure of household
- ctpay_h_ = Household’s capacity to pay
- OOPratio = Ratio of out of pocket health spending to total spending or income
- CHE30 = Catastrophic health expenditure using a 30% threshold
- poor_h_ = Poor household

#### Steps

*Step 1*: Generate food expenditure share (FES_h_) for each household by dividing the household’s food expenditure by its total expenditure:

$$ FE{S}_h=\frac{F{E}_h}{T{E}_h} $$

*Step 2:* Generate the equivalent household size (HES) for each household as: *HES* = *HS*^*β*^where HS is the household size. The two values of the coefficient β (which is a household scale multiplier) used are 0.41 and 0.56 [1]. The value of 0.56 is obtained in [12] from a regression equation based on 59 countries of the form:ln(*FE*_*h)*_ = ln(*k)* + *β* ln(*HS)* + ∑ *γ*_*i*_*country* We estimate a similar equation: ln(*FE*_*h*_*)* = ln(*k)* + *β*ln(*HS)* + ∑ *γ*_*i*_*slums* in our case. This estimation yielded a coefficient β of 0.41.

*Step 3:* Divide each household food expenditure (FE_h_) by the equivalent household size (HES) to get equivalent food expenditures (EFE_h_): $EF{E}_h=\frac{F{E}_h}{HES}$

*Step 4:* Identify the food expenditure shares of total household expenditure that are at the 45th and 55th percentile across the whole sample. Name these two variables as *FES*_*h*_*45* and *FES*_*h*_*55*. Calculate the average of food expenditure of the households in the 45th to 55th percentile range to obtain the subsistence expenditure per (equivalent) capita, which is also the poverty line (*PL*):

*PL* = *averageofEFE*_*h*_, *where FES*_*h*_45 < *EFE*_*h*_ < *FES*_*h*_55

*Step 5:* Calculate the subsistence expenditure for each household (SE_h_) as: *SE*_*h*_ = *PL* * *HES.*

*Step 6:* Compute the household’s capacity to pay (ctpay_h_): The household’s capacity to pay *(ctpay*_*h*_) is defined as the non-subsistence effective expenditures of the household. However, some households may report food expenditure that is lower than subsistence spending (SE_h_ *> FE*_*h*_); in which case, *FE*_*h*_, is used.

Thus *ctpay*_*h*_ is computed as:

$$ \begin{array}{l} ctpa{y}_h=T{E}_h-S{E}_h\; if\;S{E}_h<=F{E}_h\\ {}\kern2.04em =T{E}_h-F{E}_h\kern0.24em  if\;S{E}_h>F{E}_h\end{array} $$

*Step 7:* OOP health payments share of household capacity to pay is defined as the ratio of OOP payments to the household’s capacity to pay. That is:

$$ OO \Pr atio=\frac{OOP\; spending}{ctpa{y}_h} $$

*Step 8:* Catastrophic health expenditure (CHE):

CHE occurs when a household’s total OOP health payments equal or exceed some pre-defined percentage of its capacity to pay or non-subsistence spending. Xu et al. used a threshold of 40% [1]. In this study, the threshold is varied from 10 to 30% in the sensitivity analysis. The catastrophic health expenditure variable is constructed as a binary = 1 if household incurred catastrophic expenditure, and 0 otherwise. For example catastrophic expenditure using 30% (CHE30) as threshold would be obtained as follows:

$$ CHE30=\left[\begin{array}{l}1\; if\;OO \Pr atio>=0.3\\ {}0\; if\;OO \Pr atio<0.3\end{array}\right] $$

The coefficient β in step (2) is a scale multiplier used to adjust subsistence expenditure to account for economies of scale at the household level as its size increases. In other words, β accounts for the fact that as household size increases, subsistence expenditure increases less than proportionally.

*Step 9:* Poor household: A household is considered poor if its total spending is less than the computed subsistence expenditure:

$$ poo{r}_h=\left[\begin{array}{l}1\kern0.24em  if\;T{E}_h<S{E}_h\\ {}0\kern0.24em  if\;T{E}_h\ge S{E}_h\end{array}\right] $$

Model 1A uses household scale multiplier β = 0.41, and 45th – 55th percentile to compute subsistence expenditure. For sensitivity analysis in our study, we varied the household scale multiplier β 0.56 as in the paper by Xu. This is shown as Model 1B in Table 1.

#### Proportionality of income approach

To check the robustness of the results obtained using the above methodology, we use the income approach. In this approach we consider the OOP payments as a proportion of income (X): $\frac{OOP\; spending}{Incom{e}_h}$. Household OOP spending greater than a pre-specified fraction of their income on health is termed catastrophic. Thresholds ranging from 5% to 30% were used. The results based on this approach are presented as Model 2 in Tables 1 and 3.

**Table 3:** **Determinants of CHE based on proportionality of income approach**

	**Model 2**	
**Variable (N = 8171)**	**5%**	**10%**
**Round:**		
Round 4 (Ref)		
Round 5	1.37 ^a^(0.16^b^)**	1.27 (0.17)*
Round 6	1.49(0.19)***	1.33(0.19)**
Round 7	1.27(0.16)*	1.20(0.16)
**Slums:**		
Viwandani (Ref)		
Korogocho	0.41(0.04)***	0.30(0.03)***
Dandora	0.49(0.07)***	0.36(0.06)***
Mukuru	0.98(0.08)	0.76(0.07)***
Obunga	1.57(0.15)***	1.52(0.15)***
Nyalenda	1.40(0.12)***	1.17(0.11)*
**Income:**		
Lower tertile (Ref)		
Middle tertile	..	..
Top tertile	..	..
**Expenditure:**		
Lower tertile (Ref)		
Middle tertile	1.14(0.08)*	1.12(0.08)
Top tertile	1.65(0.12)***	1.48(0.12)***
**Livelihood:**		
Petty trade, casual (Ref)		
Formal labor	0.99(0.07)	1.05(0.08)
Own business	0.91(0.07)	1.02(0.09)
**Gender of bread winner:**		
Male	1.11(0.07)*	0.93(0.06)
**Working adult in household**	0.41(0.03)***	0.33(0.03)***
**Child under 5 years**	1.33(0.08)***	1.38(0.09)***
**Age of main income earner:**		
Age < =35 (Ref)		
35 < Age < =55	1.05(0.07)	1.07(0.8)
Age >55	1.36(0.18)**	1.57(0.22)***
**Duration of stay in slum (years)**	1.01(0.003)***	1.01(0.004)***
**Safety net**	0.78(0.05)***	0.78(0.05)***
**Shocks**	0.94(0.07)	0.93(0.08)
**Type of illness:**		
Other (Ref)		
Diarrhea	0.97(0.11)	0.97(0.12)
Fever	0.95(0.08)	0.94(0.09)
Cough	0.88(0.08)	0.90(0.09)
Headache	0.99(0.09)	0.95(0.09)
Vomiting	1.32(0.16)**	1.24(0.17)
Seizure	1.51(0.49)	1.02(0.36)
Difficult breathing	1.12(0.16)	1.16(0.19)
Measles	1.14(0.31)	1.20(0.33)
Injury	1.43(0.29)*	1.57(0.35)**
**Care sought for illness:**		
No ill person/no care sought (Ref)		
Ill/no care sought	1.17(0.16)	1.05(0.16)
Ill/care sought	1.43(0.12)***	1.22(0.11)**
**Place where care was sought:**		
Other (Ref)		
Public hospital	2.32(0.27)***	2.05(0.25)***
Public clinic	1.08(0.14)	0.78(0.12)*
Private hospital	2.31(0.43)***	1.86(0.37)***
Private clinic	1.51(0.23)***	1.27(0.22)
Mission hospital	2.59(1.03)**	2.37(1.00)**
Mission clinic	1.19(0.29)	0.95(0.28)

**Notes**: Dependent variable =1 if the household experienced catastrophic health expenditure. (^a^) reports the odds ratio. (^b^) reports the standard error. *, **, *** indicate significance at 10, 5 and 1% level respectively. Model 2: CHE is based on OOP as a proportion of household income.

Descriptive statistics of the sample and the distribution of CHE computed using various approaches are reported, followed by logistic regression analysis to identify the determinants of catastrophic health expenditure. Stata 12 (State College) was used for all analyses.

## Results

### Descriptive statistics

Table 4 shows the summary statistics of the main variables. About 23% of the households reported formal labour (operationalized as a regular employment with a steady payment) as the main source of the family income. A slightly smaller proportion (15%) owned a business. The larger majority (62%) were engaged in casual work, petty trade or were unemployed. In about 60% of the households surveyed, the main breadwinner was a woman. About three in four households had only one working adult, while 61% of all the households had children aged under five years. The average length of stay in the informal settlement was about 7.9 years. About 28% of households had a member involved in some type of social safety net such as a merry-go-round. In the last month before the survey, 17% of the households experienced some shocks such as burglary, fire, floods, mugging, eviction, property destruction or rape. Some 43% of the households reported an illness for which care was sought in the last two weeks before the survey. A not insignificant 5.3% of households had ill members but did not seek care. Headache, cough, and fever were the most common ailments reported.

**Table 4:** **Descriptive statistics for sampled households**

**Variables**	**No. of observations**	**Description**	**Mean (Standard deviation)**
Round	9447	Round 4	0.173
		Round 5	0.263 (0.44)
		Round 6	0.275 (0.45)
		Round 7	0.289 (0.45)
Slum	9447	Viwandani	0.221
		Korogocho	0.219 (0.41)
		Dandora	0.087 (0.28)
		Mukuru	0.219 (0.41)
		Obunga	0.114 (0.32)
		Nyalenda	0.141 (0.35)
Income group	9447	Lower third income group = 1	0.285
		Middle third income group = 1	0.290 (0.45)
		Top third income group = 1	0.426 (0.49)
Expenditure group	9447	Lower third spending group = 1	0.332
		Middle third spending group = 1	0.333 (0.47)
		Top third spending group = 1	0.335 (0.47)
Main Source of livelihood	9431	Other^1^ = 1	0.617
		If has formal labor (livelihood1) = 1	0.234 (0.42)
		If has own business (livelihood2) = 1	0.149 (0.36)
Gender of main bread winner	9055	Male = 1	0.402 (0.40)
		Female = 0	0.598
Working adults in household	9431	More than one working adult = 1	0.238 (0.42)
		One working adult = 0	0.763
Children under 5 years of age	9430	Household has at least one child under 5 = 1	0.614 (0.49)
		Otherwise = 0	0.386
Age of main income earner	9447	Age < = 35	0.646
		35 < Age < =55	0.268 (0.44)
		Age > 55	0.086 (0.28)
Duration of stay in the slum (years)	9353	Number of years household has stayed their current village/slum	7.88 (9.17)
Use of a social safety net by member of household	9431	Enrolled =1	0.281 (0.45)
		Otherwise = 0	0.719
Shocks	9431	If household experienced shock^2^ = 1	0.166 (0.37)
		Otherwise =0	0.834
Type of illness	9339	If suffering from diarrhea = 1;	0.064 (0.25)
		If suffering from fever =1;	0.184 (0.39)
		If suffering from cough = 1;	0.150 (0.36)
		If suffering from headache = 1;	0.151 (0.36)
		If vomiting = 1;	0.056 (0.23)
		If has convulsions = 1;	0.005 (0.07)
		If has difficulty breathing = 1;	0.035 (0.18)
		If has measles = 1;	0.008 (0.09)
		If has some injury = 1;	0.016 (0.13)
		If suffering from other^3^ = 1	Missing one
Care sought for illness	9334	No ill person, and no care sought = 1	0.516
		Ill person and no care sought = 1	0.053 (0.22)
		Ill person and care sought = 1	0.432 (0.50)
Place care was sought	9339	Care sought in public hospital =1	0.056 (0.23)
		Care sought in public clinic =1	0.067 (0.25)
		Care sought in private hospital = 1	0.019 (0.14)
		Care sought in private clinic = 1	0.038 (0.19)
		Care sought in mission hospital = 1	0.004 (0.06)
		Care sought in mission clinic = 1	0.17 (0.13)
		Care sought in other^4^ = 1	Missing one here

Notes: ^1^Other = do not have formal employment or own business, but engage for example in petty trading, casual labor or unemployed. ^2^Shock events include: fire, floods, mugging, burglary, eviction, property destruction or rape. ^3^Suffering from other type of illness. ^4^Sought other type of care or from other places for example bought over the counter drugs, from chemist, or visited an herbalist. For all categories, the minimum = 0 and the maximum = 1.

### Distribution of household expenditure

The household average expenditure for the sample was 16,754.8 KES (standard deviation 12,821.5), while the average income was 11,784.1 KES (standard deviation of 10,550; not shown). Households were on average negative savers. In other words, they are indebted. A sizeable proportion, (38.5%), of the households reported zero spending on healthcare. The average OOP health spending over the four week study period was 337.7 KES, while the average of OOP expenditure to household expenditure ratio was about 1.7% (standard deviation: 4.9). Health spending absorbed 5.0% (standard deviation: 0.39) of income, or about 2.6% (standard deviation: 0.07) of household capacity to pay (computed as described above in Step 6). Note that food was the single most important component of household spending absorbing about 42.8% of total household expenditure.

### Results of CHE computations

Table 1 shows results for Model 1A based on WHO’s Xu [1] procedure with household scale multiplier β = 0.41, and 45th -- 55th percentile. The household subsistence expenditure was 6,439.4 KES. The proportion of households classified as poor (that is whose overall household expenditure was less than the computed household subsistence expenditure) was 6.8%. The average capacity to pay was 11,549.5 KES, and declined modestly with the increase in the scale multiplier or percentile. The percentage of households classified as having catastrophic health spending decreased from 6.04% to 1.52% as the threshold was increased from 10% to 30% as expected. For sensitivity analysis, we also provide results using β = 0.56 and 45th -- 55th percentile as in Xu [1]. The results are shown in Table 1, Model 1B, and are quite similar to that obtained in Model 1A. Households classified as having catastrophic expenditure decreased from 6.09% to 1.55% as the threshold increased from 10% to 30%. The proportion of households classified as poor was 7.4%, while the average capacity to pay was 11,513.6 KES. In Model 2, Table 1, CHE is computed as a ratio of OOP to total income above some threshold. The number of households categorised as having CHE is much higher; 18% at 30% threshold to 22.8% at 10% threshold.

### Determinants of catastrophic health expenditure

In this section, we analyse the factors that determine CHE using logistic regression. Table 2 provides the logistic regression results for threshold levels ranging from 10% to 30%. This first set of regression analyses is based on the Xu [1] approach (using a household multiplier *β* = 0.41 and 45th – 55th percentile to compute subsistence expenditure in the derivation of the CHE. This is referred to as Model 1A in Table 1). A consistent set of variables emerge as significantly impacting the odds of CHE across cut-off levels, though the exact magnitude of effect does show some variation. Kisumu (Obunga and Nyalenda) slum residents were less likely to experience CHE. We found that an increase in the number of working adults in the household reduced the odds of CHE. Having two or more working adults in the household reduced the likelihood of catastrophic expenditure by at least 1.2 times (1/0.82). Also, households with a main income earner older than 55 years were at least 1.56 times more likely to experience CHE. The average number of years a household had lived in the slum appears to increase the risk of CHE. While the magnitude is small (coefficient = 1.02), it is significant across most models suggesting either a deterioration of health with time spent in the slums or a reduction in the resources available for utilizing health care services. Interestingly, we found that enrolment in an informal social safety net (such as membership in merry-go-round) reduced the risk of catastrophic spending. Households with a member enrolled in a safety net were 1.59 (1/0.63) times less likely to incur CHE.

Logically, OOP health spending would also depend on the type of illness and the type of health service sought or where health service is sought. Our results support this view. Relative to other types of illnesses, simple illness like coughs did not increase the likelihood of catastrophic expenditure. Injury however increases the likelihood of CHE. In terms of where care was sought, visiting a hospital (public or private and to a lesser extend a mission hospital) increased the chances of CHE compared to seeking remedy by purchasing drugs from a pharmacy, or over the counter; visiting a public hospital increased the likelihood of CHE by at least 3.9 times.

We also considered the effect of seeking healthcare. Compared to households where no members felt sick or where no care was sought, those with members requiring medical care for illness faced an increased likelihood of CHE by at least 1.7 times.

Additional robustness tests were carried out. Table 3 provides the logistic regression results for the case when CHE is computed based on proportionality of income (Model 2). The analyses provide a set of results that are slightly different from that obtained from Models 1A. For example, residents of Kisumu slum (Obunga) had a higher risk of experiencing CHE. Source of livelihood variables (formal employment and own business) did not show up as significant factors influencing CHE in Model 2.

Overall, however, a small core set of variables (expenditure, number of working adults in the household, age of main income earner > 55 years, obtaining care from public or private hospital) emerged as significantly impacting the odds of CHE across both models and threshold levels presented in Tables 2 and 3. The exact magnitude of effect did show some variation across the different models.

## Discussion

Our results indicate that the incidence of CHE is sensitive to the method and thresholds used. Using the WHO method yields the lower incidence of CHE with a range of 1.52% to 6.15% corresponding to a threshold of 30% to 10%. Using the threshold of 40% common in the literature would yield an incidence lower than 1.52%, i.e. almost no household in the slum incurs catastrophic health expenditures. On the other hand, the proportionality of income method yields the highest incidence: 18.46% to 28.38% of households faced CHE corresponding to the thresholds of 30% to 5%.

The source of those differences lies in the denominator as the numerator is the same across methods. The WHO method relates the OOP to the capacity to pay calculated as total expenditures adjusted for the subsistence expenditures on food, and household size. On the other hand, the OOP/ income ratio relates the OOP to the household’s income. In our sample, the average household’s expenditure is 42% higher than its income, so that despite the adjustment down to the expenditure, methods with capacity to pay using expenditures will yield lower estimates of CHE.

It is noteworthy that the incidence of CHE in our study—using ratio of OOP to income—is higher than that found in a nationwide study representative of Kenya, which found that 9.5% and 8.7% of households respectively faced CHE when using thresholds of 25% and 40%, respectively [20].

Households in our sample are poor as illustrated by the fact that food consumes about half the income [25] and a large proportion of residents are not food secure [27]. Indeed, for about 56% of the households in our sample, food spending is below what is considered as needed to provide adequate calorie intake according to the government of Kenya’s estimate. As food, shelter, and other necessities exhaust the bulk of the household’s income, little is left for other items of expenditures such as health care. It is possible that informal settlements residents are not able to afford the care they may need, implying that the low incidence of CHE measured with the WHO method and other method based on capacity to pay may be due to the fact that poor households forgo healthcare. These results illustrate inequity in the access to care when payment for the care is out of pocket.

These results may be linked to the fact that households in the top income tertile have reduced odds of incurring CHE relative to those in the lower tertile although this effect is not always measured with precision. Similar results have been found in Kenya [20], and in Burkina Faso [28]. It appears that higher income protects against CHE. The fact that the coefficient is not always statistically significant despite the large magnitude may be due to the small sample size.

Concerning the important determinants of CHE in urban slums, we find that in all models, a higher number of working adults in a household and membership in a social safety net reduced the risk of catastrophic expenditure. Conversely, a main income earner older than 55 years, the longer the number of years resident in the slum, and the seeking care in a hospital (whether public or private) were each associated with a higher risk of CHE.

Having two or more working adults in the household reduced the likelihood of catastrophic expenditure probably by increasing the household’s income more than its spending. This result is intuitive though it contradicts earlier findings [17]. A possible explanation may be the age of the workers. In our sample 64% of the main breadwinners are relatively young - 35 years old or younger. Their partners are likely to be about the same age. Thus, their earned income tends to increase relatively more than the out-of-pocket expenditure. The finding that households with a head older than 55 years are more likely to experience CHE support results of other studies [17] [18]. A number of studies have tackled the effect of health insurance enrolment and or availability on catastrophic expenditure [15,18,29]. In general, the findings in the literature support our results related to membership of social safety nets in the slums and the risk of CHE. Households enrolled in some type of health insurance are generally found to be at a lower higher risk of CHE.

Variables related to the main source of family income affected the household’s likelihood of experiencing catastrophic spending. Having a formal employment or owning a business both reduce the risk of CHE. In a recent study that analysed the ways households cope with financial shortfalls in Nairobi slums [25], formal employment and owning a business reduced the chances of using negative coping strategies such as not eating enough meals or withdrawing children from school.

The effect of healthcare service utilization probably reflects the severity of the disease/condition, the higher cost of getting a service in a hospital and the nature of service needed. Compared to seeking remedy by purchasing drugs over the counter from a pharmacy, visiting a public hospital increased the likelihood of CHE by almost four times. Our results corroborate existing literature. A severe ailment or injury that requires inpatient care has been found to increase the likelihood of catastrophic spending [17], as well as the use of inpatient service especially in private hospital [3]. The finding that an illness by itself, even when no care was sought, increased the likelihood of a household experiencing CHE probably reflects the loss of income caused by work days due to sickness (and reduced expenditures) in a slum population largely engaged in casual employment that pays per day worked.

This study has some limitations. The income and expenditures data is self-reported and not verifiable from other sources. Also, additional non-cash income sources were not included in the household income data collected although as these sources are likely to be negligible in urban informal settlements. The recall period for expenditures on healthcare was three months, much longer than two weeks which was shown to be associated with the best recall; but much shorter than one year which has been associated with a worse recall of expenditures on health [30]. We cannot ascertain that possible inaccuracies in recall occur similarly for income or expenditures. Thus, we cannot exclude measurement errors in estimating OOP, income and expenditures among population where the majority do not have regular and steady source of income.

Despite those limitations, this study provides some useful insights into the challenges of health expenses among the urban poor in Kenya and should serve as a basis for more detailed investigations across the many slums in Kenya and sub-Saharan Africa. Our study made several contributions. It examined the determining factors of CHE in Kenya among low-income residents of informal settlements (slums) using a unique data set. Such focus is rare in the literature but justified by the fact that in Kenya about 60% of the residents of cities live in slum or slum-like conditions [26]. In addition, our study used a variety of methods proposed in the literature for robustness checks. No previous studies have applied the WHO method [1] to study CHE in Kenya. This method is preferred considering the difficulty in measuring income especially among informal settlement dwellers. However, users need to be aware that this method may indicate low prevalence of catastrophic expenditures among poor households who have little funds for healthcare.

## Conclusion

Our study indicates that the proportion of households facing catastrophic health payment varies according to the method used. As expected the incidence decreases as the threshold increases. However, the proportions are not negligible especially considering the study was conducted among residents of slum areas where the vast majority of residents are poor or vulnerable to poverty. The small proportion of CHE found with methods related to capacity to pay are likely to reflect the fact that in those slum areas, poor households have little income left after other expenditures to spend on healthcare and probably forgo the care needed. This raises an inequity question as to what happens to the poorest of the poor who cannot afford health care when a health crisis starts. The analysis of determinants of CHE indicates that a stable livelihood acts as a bulwark against CHE and seeking care from public hospitals still proves catastrophic for households. Solutions to catastrophic health expenditures go beyond improved job opportunities and availability of publicly provided health care in these deprived settlements. The results of this study call for an insurance mechanism that is equitable, affordable and inclusive to the poor urban slums residents. We also found that enrolment in a social safety net, such as merry-go-round, reduces the likelihood of CHE. This suggests that small scale insurance programs operated at community level may be an effective strategy for protecting the low-income households against impoverishment due to health expenditures.
